# Medikamenteninduzierter Lichen planus mit Ansprechen auf Upadacitinib

**DOI:** 10.1007/s00105-025-05517-w

**Published:** 2025-06-04

**Authors:** Lea Victoria Goerdt, Giulnara Grygorian, Michael P. Schön, Rotraut Mössner

**Affiliations:** Klinik für Dermatologie, Venerologie und Allergologie, Universitätsmedizin Göttingen, Robert-Koch-Str. 40, 37075 Göttingen, Deutschland

Der Lichen planus (LP) ist eine subakute oder chronische, nicht ansteckende, entzündliche Erkrankung der Haut und der Schleimhäute. Sie kann durch Medikamente ausgelöst werden, wobei zwischen der ersten Einnahme des Medikaments und der Manifestation der Krankheit einige Wochen bis mehrere Monate liegen können [1].

## Fallbeispiel

**Abb. 1:**
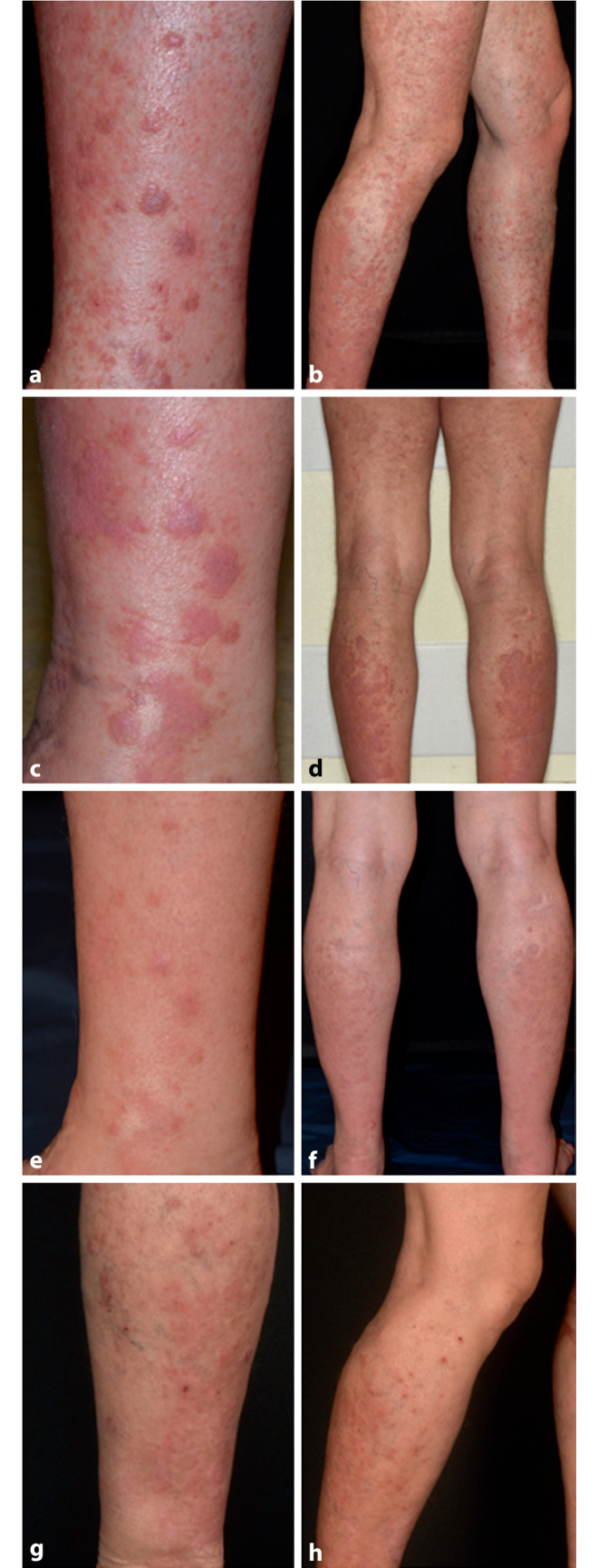
**a**, **b** Flachspitzige erythematöse bis violette Papeln und Plaques an den Unterschenkeln nach 11-monatiger Therapie mit Dupilumab. **c**, **d** Unterschenkel einen Monat nach Absetzen von Dupilumab. **e**, **f** Unterschenkel nach einem Monat unter Therapie mit Upadacitinib und 2 Monate nach Absetzen der Therapie mit Dupilumab. **g**, **h** Klinisches Bild 7 Monate nach Absetzen der Upadacitinib-Therapie mit ekzematösen Läsionen an den rechten Unterschenkeln

**Abb. 2:**
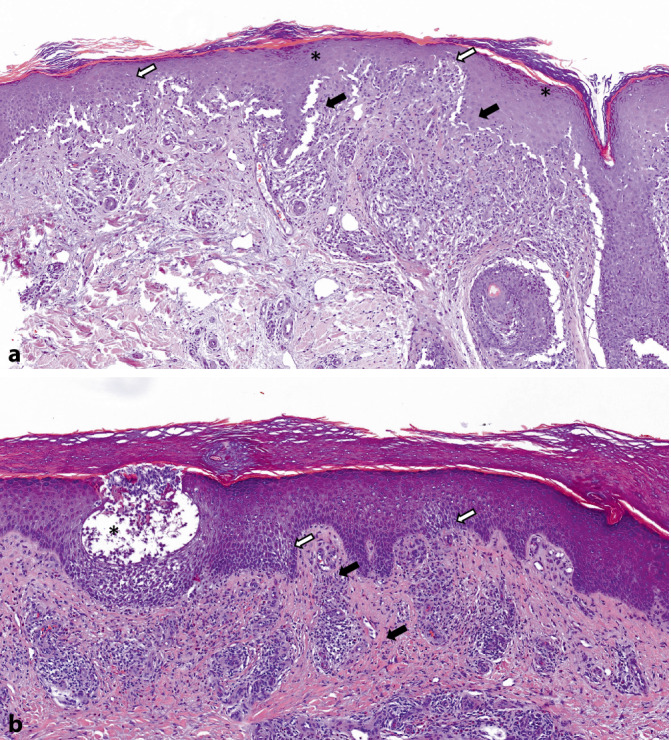
Hautläsionen unter verschiedenen Therapien. **a** Lichenoide Dermatitis (Lichen planus) mit sägezahnförmiger Akanthose, Orthohyperkeratose, dyskeratotischen Keratinozyten (*offene Pfeile*), fokaler Hypergranulose (*Sternchen*), subepidermaler Spaltbildung (*gefüllte Pfeile*) und bandförmigem lymphozytärem Hautinfiltrat. **b** Ekzematöse Läsion mit unregelmäßiger Akanthose, Spongiosa (*offene Pfeile*) mit spongiotischen Pusteln (*Sternchen*) und Hyperkeratose sowie dermalem perivaskulärem und interstitiellem, überwiegend lymphozytärem Infiltrat, ektatischen Blutgefäßen (*gefüllte Pfeile*) und einigen Erythrozytenextravasaten. Maßstabsbalken = 100 µm

Ein 71-jähriger Mann stellte sich mit einer seit Langem bestehenden, schweren atopischen Dermatitis vor. Die durchgeführten Therapien – einschließlich topischer Kortikosteroide und verschiedener Phototherapien – waren nicht in der Lage, die Hautkrankheit hinreichend zu kontrollieren. Daher entschied man sich, eine Systemtherapie mit Dupilumab (zunächst 2‑mal 300 mg s.c., dann 300 mg s.c. alle 2 Wochen) einzuleiten. Nach 11-monatiger Behandlung mit Dupilumab waren die ekzematösen Hautläsionen tatsächlich fast verschwunden. Einige Wochen vor der Vorstellung bei uns waren jedoch neue, flache, erythematöse bis bläulich-livide, lichenoide Papeln und Plaques mit starkem Juckreiz v. a. am unteren Rücken und an Armen und Beinen aufgetreten (Abb. 1a, b). Die Schleimhäute waren nicht betroffen. Die Anamnese war ansonsten unauffällig mit Ausnahme der Einnahme von Simvastatin, die eine Woche vor Auftreten der lichenoiden Läsionen begonnen wurde. Die histopathologische Untersuchung der Biopsien vom unteren Rücken und vom linken Unterschenkel zeigten eine fokale kompakte Orthohyperkeratose, Akanthose und eine ausgeprägte Verbreiterung des Stratum granulosum sowie ein bandförmiges, lymphozytäres Infiltrat an der dermoepidermalen Junktionszone mit Exozytose in die Epidermis im Sinne einer Interfacedermatitis, vereinbar mit der Diagnose eines Lichen planus (LP) (Abb. 2a). In der Histologie zeigte sich aktuell kein Hinweis auf eine lichenoide Mycosis fungoides. Da aufgrund der Anamnese der Verdacht bestand, dass der LP medikamenteninduziert sein könnte, wurde Simvastatin abgesetzt und eine UVA1-Phototherapie in Kombination mit topischen Glukokortikosteroiden begonnen. Da sich die Hautläsionen nach einem Monat weiter progredient zeigten, wurde Dupilumab ebenfalls abgesetzt und der Patient einen Monat später mit Upadacitinib (15 mg/Tag) behandelt (Abb. 1c, d). Die lichenoiden Läsionen besserten sich unter Therapie mit Upadacitinib innerhalb von 4 Wochen erheblich (Abb. 1e, f) und klangen innerhalb von 2 weiteren Monaten fast vollständig ab. Upadacitinib wurde daraufhin abgesetzt, und nach einem weiteren Monat wurde eine Behandlung mit Atorvastatin begonnen. Sieben Monate nach Absetzen der Upadacitinib-Therapie stellte sich unser Patient mit ekzematösen Läsionen an den Unterschenkeln, Ellenbogen und am oberen Rumpf vor (Abb. 1g, h). Der klinische Verdacht auf ein Rezidiv der atopischen Dermatitis wurde durch die histopathologische Untersuchung bestätigt (Abb. 2b). Daraufhin wurde eine topische Therapie mit Glukokortikosteroiden eingeleitet.

Bei unserem Patienten stellt sich die Frage, ob es sich um einen spontan aufgetretenen oder um einen medikamenteninduzierten Lichen planus (LP) handelt. Für eine Auslösung durch ein Medikament kommen bei unserem Patienten sowohl Simvastatin als auch Dupilumab infrage. Simvastatin scheint als Auslöser weniger wahrscheinlich zu sein, da die Latenzphase nach Beginn der Einnahme von Simvastatin bis zum Auftreten des LP kurz war, die Läsionen nach dem Absetzen persistierten und nach der Einnahme eines verwandten Arzneimittels keine neuen Läsionen auftraten [5, 6]. Andererseits wurden bereits mehrere Fälle von de novo induzierten lichenoiden Eruptionen nach einer Dupilumab-Therapie der atopischen Dermatitis publiziert, sodass wir auch bei unserem Patienten von einem pathogenetischen Zusammenhang ausgehen [2–4].

## Diskussion

Obwohl der LP nicht selten ist, ist die Pathogenese des LP noch nicht vollständig verstanden [7, 8]. In der Literatur finden sich zunehmend Hinweise darauf, dass es sich beim LP um eine T‑Zell-vermittelte Autoimmunerkrankung handelt, die durch exogene Noxen wie Medikamente, Viren oder Kontaktallergene ausgelöst werden kann. Epidermale Selbstantigene können dann zur Aktivierung von T‑Zellen und zur Entwicklung einer lichenoiden Entzündung führen, wobei hier insbesondere die Typ-I-Interferone eine zentrale Rolle spielen, indem sie die Expression von IP10/CXCR10 erhöhen und Effektorzellen über CXCR3 rekrutieren [9, 10]. Zu den Medikamenten, die mit der Entwicklung von LP in Verbindung gebracht werden, gehören zielgerichtete Therapien wie Tumornekrosefaktor-α-Antagonisten sowie Immuncheckpointinhibitoren und neuerdings Dupilumab [2, 11, 12]. Bemerkenswert ist, dass sich in einigen Fällen von mit Dupilumab behandelten Patienten mit atopischer Dermatitis der LP in den ehemals ekzematösen Bereichen entwickelte, was auf eine Umstellung der lokalen Immunantwort hindeutet [3, 4, 12]. Es wird angenommen, dass dieser Prozess von der Aktivierung der Januskinase 2 (JAK 2) und des STAT1 („signal transducer and activator of transcription 1“)-Signalwegs gesteuert wird, sodass man davon ausgeht, dass JAK-1/2-Inhibitoren Keratinozyten vor dieser Art von zellvermittelten zytotoxischen Reaktionen schützen [13].

Überraschenderweise gibt es jedoch auch mehrere Berichte, die eine erfolgreiche Behandlung von LP [14, 15] und von LP-Pemphigoid [16, 17] mit Dupilumab beschreiben. Diese widersprüchlichen Befunde legen nahe, dass die IL(Interleukin)-4/IL-13-Immunachse eine janusartige Rolle bei der Verschiebung des Gleichgewichts des Immunsystems in Richtung oder gegen eine lichenoide Entzündung spielen kann, abhängig von bisher unbekannten Einflussfaktoren. In unserem Fall sowie bei anderen durch Dupilumab induzierten lichenoiden Hautreaktionen vermuten wir, dass die Behandlung mit Dupilumab zu einer Herunterregulierung der Th2-Zellaktivität geführt hat, was eine Verschiebung hin zu einer Th1-vermittelten Immunantwort und damit eine CD8-getriebene zytotoxische Hautentzündung begünstigt hat.

Auf dieser pathoimmunologischen Grundlage behandelten wir unseren Patienten mit Upadacitinib. Upadacitinib ist ein reversibler Januskinase(JAK)-Inhibitor, der selektiv für JAK 1 gegenüber JAK 2 und JAK 3 ist (Tab. 1). Die Tatsache, dass Upadacitinib gegen den lichenoiden Ausschlag bei unserem Patienten wirksam war, obwohl die Ergebnisse von Shao et al. darauf hindeuten, dass JAK 2 der Hauptverursacher bei Lichen planus sein könnte [13], lässt vermuten, dass sowohl JAK 2 als auch JAK 1 an der lichenoiden Entzündung beteiligt sind [18] oder dass Upadacitinib immer noch breit genug wirkt, um auch JAK 2 ausreichend zu hemmen.

Das gute Ansprechen des LP auf Upadacitinib bei unserem Patienten steht im Einklang mit einem früheren Bericht über eine ausgezeichnete therapeutische Wirkung von Upadacitinib und anderen JAK-Inhibitoren bei kutanem und mukosalem Lichen planus [19].

**Tab. 1:** JAK(Januskinase)-Inhibitoren: ihre Selektivität und Effizienz in der Therapie des Lichen planus

JAK-Inhibitor	Selektivität	Vollständige Remission	Teilweise Remission	Keine Remission	Studiendetails
Baricitinib	JAK 1/2	25 % (4/16)	31,3 % (5/16)	43,8 % (7/16)	Abduelmula et al. und Hwang et al. [20, 21]
Tofacitinib	JAK 1/3	10 % (3/30)	60 % (18/30)	30 % (9/30)	Abduelmula et al. [20]
Ruxolitinib	JAK 1/2	16,7 % (2/12)	83,3 % (10/12)	0 %	Abduelmula et al. [20]
Upadacitinib	JAK 1	100 % (2/2)	0 %	0 %	Abduelmula et al. [20]
Deucravacitinib	TYK2	0 %	100 % (1/1)	0 %	Ball et al. [22]

*TYK2* Tyrosinkinase 2

## Fazit für die Praxis

- Zusammenfassend lässt sich festhalten, dass Dupilumab in die Liste der Arzneimittel aufgenommen werden muss, die einen Lichen planus (LP) auslösen können.
- Dieser medikamenteninduzierte LP spricht jedoch offenbar gut auf eine Behandlung mit Upadacitinib an.
